# Supplementary material for: Evaluating large language models for accuracy incentivizes hallucinations
Source: Nature. 2026 Apr 22;653(8116):1047–51. doi: 10.1038/s41586-026-10549-w (PMC13216060; doi:10.1038/s41586-026-10549-w)
Supplement: Supplementary file 1 — This Supplementary Information file contains Proof of Theorem 3, Proofs of Corollary 1 and Theorem 4, and additional references. [file 41586_2026_10549_MOESM1_ESM.pdf]

nature portfolio

https://doi.org/10.1038/s41586-026-10549-w

Supplementary information

Evaluating large language models for accuracy incentivizes hallucinations

In the format provided by the authors and unedited

Supplementary Information: Evaluating large language models for accuracy incentivizes hallucinations

Proof of Theorem 3

We begin by reviewing the Good-Turing (GT) estimator of missing mass[1] and its guarantees[2]. In that setting, N iid samples $s \sim \nu^N$ are drawn from distribution ν over set $\mathcal{S}$—abstentions are not a consideration. The missing mass is the probability that a new example drawn from ν would not be in the training sample s, and the estimate GT is the fraction of training samples that occur exactly once. We first state the prior guarantees and then adapt them to our setting with abstentions. A guarantee of McAllester and Ortiz[2] can be stated as:

Corollary 2. [2] *Let $s \sim \nu^N$ be N iid samples from distribution ν over set $\mathcal{S}$. Let $M := \Pr_{x\sim\nu}[x \notin s]$ and* GT *be the fraction of samples that occur exactly once. For any $\gamma \in (0, 1]$:*

$$\Pr_{s\sim\nu^N}\left[|M - \mathrm{GT}| \le \frac{1}{N} + 2.42\sqrt{\frac{\ln(4/\gamma)}{N}}\right] \ge 1 - \gamma.$$

Proof. Let $\overline{\mathrm{GT}} := \mathbb{E}[\mathrm{GT}]$ and $\overline{M} := \mathbb{E}[M]$. The corollary follows by combining concentration bounds on M and GT. First Theorem 1 of prior work[3] shows:

$$\overline{\mathrm{GT}} - \overline{M} \in [0, 1/N]$$

Then, Theorems 10 and 16[2] imply that with probability $\le \exp(-N\varepsilon^2)$, M will deviate from $\overline{M}$ by more than ε in either direction, together, by the union bound giving for $\varepsilon := \sqrt{\frac{\ln(4/\gamma)}{N}}$,

$$\Pr_{s\sim\nu^N}\left[|M - \overline{M}| \ge \sqrt{\frac{\ln(4/\gamma)}{N}}\right] \le \frac{\gamma}{4} + \frac{\gamma}{4} = \frac{\gamma}{2}.$$

Following prior work[3] Lemma 13, McDiarmid's inequality[4] directly implies the convergence of GT, since changing any one example can change GT by at most $2/N$. Hence,

$$\Pr_{s\sim\nu^N}\left[|\mathrm{GT} - \overline{\mathrm{GT}}| \ge \sqrt{\frac{2\ln(4/\gamma)}{N}}\right] \le 2\exp\left(-\frac{2 \cdot \frac{2\ln(4/\gamma)}{N}}{4/N}\right) = \frac{\gamma}{2}.$$

Combining these three displayed equations, gives, by the union bound,

$$\Pr_{s\sim\nu^N}\left[|\mathrm{GT} - M| \ge \frac{1}{N} + (1 + \sqrt{2})\sqrt{\frac{\ln(4/\gamma)}{N}}\right] \le \frac{\gamma}{2} + \frac{\gamma}{2} = \gamma.$$

Finally, the corollary follows from $1 + \sqrt{2} \le 2.42$. □

We now extend this to the case of an abstention response $\bot$ which is not counted in sr. Specifically, we say a query c is *answered* in the training data if there is a training example $(c^{(i)}, r^{(i)})$ with $c^{(i)} = c$ and $r^{(i)} \neq \bot$, and *unanswered* otherwise. Let

$$\mathcal{U} := \mathcal{C} \setminus \{c^{(i)} \mid i \leq N, r^{(i)} \neq \bot\}$$

denote the set of unanswered queries. Of course, by memorizing a_c for answered queries, one can achieve perfect accuracy classifying the answered queries. We extend Turing's Missing Mass (MM) estimate to abstentions as follows:

$$\mathrm{MM} := \Pr_{(c,r)\sim p}[c \in \mathcal{U} \wedge r \neq \bot].$$

13 We similarly use Corollary 2 to show that sr is a good estimator of MM:

Lemma 1. *For all N, $\gamma \in (0, 1]$:*

$$\Pr\left[\ |\mathrm{MM} - \mathrm{sr}| \leq 4.42\sqrt{\frac{\ln(5/\gamma)}{N}}\ \right] \geq 1 - \gamma.$$

Proof. The only difference between our MM-sr and the standard M-GT is that we ignore abstentions. To adapt the previous bounds, consider the sample s which is derived by replacing all $x = (c, \bot)$ with simply $x = \bot$ for any c, but otherwise leaving x unchanged. This collapses all IDK responses into identical examples. Thus GT may count at most one extra singleton compared to sr,

$$\mathrm{GT} - \mathrm{sr} \in \left\{0, \frac{1}{N}\right\}.$$

The above substitution induces a distribution ϕ where $\phi(\bot) = \sum_c \mu(c)p(\bot \mid c)$ is the probability of abstaining. Similarly, we have $M - \mathrm{MM} \in \{0, \phi(\bot)\}$ with $M - \mathrm{MM} = \phi(\bot)$ if $\bot \notin s$, which happens with probability $(1 - \phi(\bot))^N$. But we also have $(1 - \phi(\bot))^N \leq \gamma/5$ if $\phi(\bot) \geq \frac{1}{N}\ln\frac{5}{\gamma}$. Hence, regardless of the value of $\phi(\bot)$,

$$\Pr\left[M - \mathrm{MM} \in \left[0, \frac{1}{N}\ln\frac{5}{\gamma}\right]\ \right] \geq 1 - \frac{\gamma}{5}.$$

14 Combining the above two displayed equations gives,[1]

$$\Pr\left[\left|(M - \mathrm{GT}) - (\mathrm{MM} - \mathrm{sr})\right| \leq \frac{1}{N}\ln\frac{5}{\gamma}\right] \geq 1 - \frac{\gamma}{5}. \tag{5}$$

Corollary 2 at $\frac{4}{5}\gamma$ gives,

$$\Pr\left[\ |M - \mathrm{GT}| \leq \frac{1}{N} + 2.42\sqrt{\frac{\ln(5/\gamma)}{N}}\ \right] \geq 1 - \frac{4}{5}\gamma.$$

[1]This follows from the fact that both $A := M - \mathrm{MM}$ and $B := \mathrm{GT} - \mathrm{sr}$ are non-negative. If $0 \leq A \leq \frac{1}{N}\ln\frac{5}{\gamma}$ and $0 \leq B \leq \frac{1}{N}$, because $\frac{1}{N} \leq \frac{1}{N}\ln\frac{5}{\gamma}$, the larger of the two upper bounds is $\frac{1}{N}\ln\frac{5}{\gamma}$, so $|A - B| \leq \frac{1}{N}\ln\frac{5}{\gamma}$.

Combining with Eq. (5) gives, by the union bound and triangle inequality,

$$\Pr\left[\ |\mathrm{MM}-\mathrm{sr}| \le \frac{1}{N}\ln\frac{5}{\gamma}+\frac{1}{N}+2.42\sqrt{\frac{\ln(5/\gamma)}{N}}\ \right] \ge 1-\gamma.$$

Finally, the lemma follows from the fact that for $z := \frac{2}{N}\ln\frac{5}{\gamma} \ge \frac{1}{N}\ln\frac{5}{\gamma}+\frac{1}{N}$, we have $z \le \sqrt{z}$ as long as $z \le 1$ (otherwise the Lemma holds trivially because the bound is > 2). □

Lemma 2. *For any $N \ge 1$, $\gamma \in (0,1]$, and any algorithm outputting $\hat{p}$,*

$$\Pr\left[2\ \mathrm{err}_{\mathrm{iiv}} \ge \mathrm{sr} - \frac{6\ln(3N/\gamma)}{\sqrt{N}}\right] \ge 1-\gamma.$$

Proof. By Lemma 1,

$$\Pr\left[\ |\mathrm{MM}-\mathrm{sr}| \le 4.42\sqrt{\frac{\ln(10/\gamma)}{N}}\ \right] \ge 1-\frac{\gamma}{2}.$$

Note that $\sqrt{\ln(10/\gamma)} \le \ln(3N/\gamma)$ for $N \ge 2$ (and the lemma holds trivially for $N = 1$). Also, $\sqrt{2}+4.42 \le 6$. Hence, it suffices to show that,

$$\Pr\left[2\ \mathrm{err}_{\mathrm{iiv}} \ge \mathrm{MM} - \sqrt{\frac{2}{N}}\ln\frac{3N}{\gamma}\right] \ge 1-\frac{\gamma}{2}.$$

Let $\zeta := \ln(3N/\gamma)/N$ and the probability of each query appearing with an answer (not $\perp$) according to p to be:

$$\mu'(c) := \mu(c)\alpha_c,$$

so $\mu'(c) = p(c, a_c)$ once a_c is selected. Also note that $\mathrm{MM} = \sum_{c\in\mathcal{U}} \mu'(c)$. The lemma will thus follow from the following two inequalities:

$$\Pr\left[\forall c \in \mathcal{U}\ \mu'(c) \le \zeta\right] \ge 1-\frac{\gamma}{3} \tag{6}$$

$$\Pr\left[2\,\mathrm{err}_{\mathrm{iiv}} \ge \mathrm{MM} - \sqrt{\frac{2}{N}}\ln\frac{3N}{\gamma}\ \middle|\ \forall c \in \mathcal{U}\ \mu'(c) \le \zeta\right] \ge 1-\frac{\gamma}{6}. \tag{7}$$

The $\mu'(c) \le \zeta$ condition will enable us to use Hoeffding bounds. For Eq. (6), note that there are $\le 1/\zeta$ queries c with $\mu'(c) \ge \zeta$. For each of these queries, the probability $c \in \mathcal{U}$ is at most $(1-\zeta)^N$. Hence, by the union bound,

$$\Pr\left[\exists c \in \mathcal{U}:\ \mu'(c) > \zeta\right] \le \frac{1}{\zeta}(1-\zeta)^N \le \frac{1}{\zeta}e^{-\zeta N} = \frac{N}{\ln(3N/\gamma)}\frac{\gamma}{3N} \le \frac{\gamma}{3},$$

which is equivalent to Eq. (6). We now move on to establish Eq. (7).

Let the indicator $\mathbb{1}[\phi]$ to denote 1 if predicate ϕ holds and 0 otherwise. The error $\mathrm{err}_{\mathrm{iiv}}$ is at least its error summed over $c \in \mathcal{U}, r \in \mathcal{R}_c$, of course, which by definition of D is,

$$\begin{aligned}
\mathrm{err}_{\mathrm{iiv}} &\geq \frac{1}{2}\sum_{c\in\mathcal{U}} \mu(c)\alpha_c \mathbb{1}[\hat{f}(c,a_c) = -] + \frac{1}{2}\sum_{c\in\mathcal{U}} \mu(c) \sum_{r\in\mathcal{R}_c\setminus\{a_c\}} \frac{\mathbb{1}[\hat{f}(c,r) = +]}{|\mathcal{R}_c| - 1} \\
&\geq \frac{1}{2}\sum_{c\in\mathcal{U}} \mu'(c)\mathbb{1}[\hat{f}(c,a_c) = -] + \frac{1}{2}\sum_{c\in\mathcal{U}} \mu'(c) \sum_{r\in\mathcal{R}_c\setminus\{a_c\}} \frac{\mathbb{1}[\hat{f}(c,r) = +]}{|\mathcal{R}_c| - 1} \\
&= \sum_{c\in\mathcal{U}} \mu'(c)\gamma_c \text{ for } \gamma_c := \frac{1}{2}\left(\mathbb{1}[\hat{f}(c,a_c) = -] + \sum_{r\in\mathcal{R}_c\setminus\{a_c\}} \frac{\mathbb{1}[\hat{f}(c,r) = +]}{|\mathcal{R}_c| - 1}\right)
\end{aligned}$$

Thus $\mathrm{err}_{\mathrm{iiv}} \geq \sum_{c\in\mathcal{U}} \mu'(c)\gamma_c$ with γ_c define above, and it is not difficult to see that $\gamma_c \in [0,1]$. (The $\mu'(c) \leq \zeta$ condition will enable us to apply Hoeffding bounds to $\sum \mu'(c)\gamma_c$.) Thus instead of Eq. (7) it suffices to show,

$$\Pr\left[2\sum_{c\in\mathcal{U}} \mu'(c)\gamma_c \geq \mathrm{MM} - \sqrt{\frac{2}{N}}\ln\frac{3N}{\gamma} \;\middle|\; \forall c \in \mathcal{U}\ \mu'(c) \leq \zeta\right] \geq 1 - \frac{\gamma}{6}. \tag{8}$$

Now for the key trick: because the algorithm's output is independent of a_c for unseen $c \in \mathcal{U}$, one can equivalently imagine the a_c's being selected for unseen $c \in \mathcal{U}$ only *after* running the algorithm on the training data to select $\hat{p}$ which determines $\hat{f}$. Thus, let us suppose that a_c will later be chosen for $c \in \mathcal{U}$ but that the training data and thus $\hat{f}$ are *already fixed*.

Then, we observe that $\mathbb{E}[\gamma_c] = 1/2$ because each $r \in \mathcal{R}_c$ contributes $1/2|\mathcal{R}_c|$ to this expectation regardless of whether it is $\hat{f}(c,r) = \pm$. This gives $\mathbb{E}[\sum_c \mu'(c)\gamma_c] = \mathrm{MM}/2$ since $\mathrm{MM} = \sum_c \mu'(c)$. Finally, we can apply the Hoeffding bound to $\sum_c \mu'(c)\gamma_c$ since $\mu'(c)\gamma_c$ are independent random variables each in $[0, \mu'(c)]$. The bound depend on,

$$\sum_{c\in\mathcal{U}} (\mu'(c))^2 \leq \max_{c\in\mathcal{U}} \mu'(c) \sum_{c\in\mathcal{U}} \mu'(c) \leq \max_{c\in\mathcal{U}} \mu'(c) \leq \zeta \text{ if } \forall c \in \mathcal{U}\ \mu'(c) \leq \zeta.$$

The Hoeffding bound thus gives,

$$\Pr\left[\sum \mu'(c)\gamma_c \leq \frac{\mathrm{MM}}{2} - \sqrt{\frac{\zeta\ln(6/\gamma)}{2}} \;\middle|\; \forall c \in \mathcal{U}\ \mu'(c) \leq \zeta\right] \leq \frac{\gamma}{6},$$

which implies Eq. (8) since $\sqrt{2\zeta\ln(6/\gamma)} = \sqrt{2\ln(3N/\gamma)\ln(6/\gamma)/N} \leq \ln(3N/\gamma)\sqrt{2/N}$ (using $\ln(6/\gamma) \leq \ln(3N/\gamma)$ for $N \geq 2$ and again the lemma holds trivially for $N = 1$). □

We now prove Theorem 3.

Proof of Theorem 3. The following more general lower bound, for any $\gamma \in (0,1]$, follows directly from Theorem 2, with $\max_c |\mathcal{V}_c| = 2$, and Lemma 2. Specifically, with probability $\geq 1 - \gamma$:

$$\mathrm{err} \geq \mathrm{sr} - \frac{2}{\min_c |\mathcal{E}_c|} - \frac{6\ln(3N/\gamma)}{\sqrt{N}} - \delta.$$

For $\geq 99\%$ probability at $\gamma = 0.01$, we use the simplification that $6\ln(3N/\gamma) \leq 35 + 6\ln N$. Now let $L := \max_c |\mathcal{E}_c|$.

For the upper bound, we now show that there is an efficient algorithm outputting calibrated $\hat{p}$ (so $\delta = 0$), and with probability $\geq 1 - \gamma$,

$$\text{err} \leq \text{sr} - \frac{\text{sr}}{L+1} + 5\sqrt{\frac{\ln(5/\gamma)}{N}}.$$

The 99% probability bound in the theorem follows from $5\sqrt{\ln(500)} \leq 13$.

The calibrated language model learning algorithm memorizes a_c for (c, a_c) seen in the training data and agrees perfectly with p on those $c \notin \mathcal{U}$ seen in the training data. For the unseen $c \in \mathcal{U}$, it abstains with the correct probability $1 - \alpha_c$ but otherwise is uniformly random over $\mathcal{R}_c$:

$$\hat{p}(r \mid c) := \begin{cases} 1 - \alpha_c & \text{if } r = \bot \\ \alpha_c & \text{if } c \notin \mathcal{U}, r = a_c \\ \alpha_c / |\mathcal{R}_c| & \text{if } c \in \mathcal{U}, r \in \mathcal{R}_c \\ 0 & \text{otherwise.} \end{cases}$$

It is easy to see that, for this $\hat{p}$,

$$\text{err} = \sum_{c \in \mathcal{U}} \mu(c) \frac{\alpha_c}{|\mathcal{R}_c|}(|\mathcal{R}_c| - 1) \leq \sum_{c \in \mathcal{U}} \mu(c) \alpha_c \frac{L}{L+1} = \text{MM} \frac{L}{L+1}.$$

Finally, by Lemma 1

$$\Pr\left[|\text{MM} - \text{sr}\,| \leq 5\sqrt{\frac{\ln(5/\gamma)}{N}} \right] \geq 1 - \gamma.$$

These imply,

$$\Pr\left[\text{err} \leq \frac{L}{L+1}\,\text{sr} + 5\sqrt{\frac{\ln(5/\gamma)}{N}} \right] \geq 1 - \gamma,$$

as needed. It only remains to show that $\delta_z = 0$ for all $z \in [0, 1]$. By definition of δ_z,

$$\begin{aligned} \delta_z &= \left| \Pr_{(c,r)\sim\hat{p}}[\hat{p}(r \mid c) > z] - \Pr_{(c,r)\sim p}[\hat{p}(r \mid c) > z] \right| \\ &= \left| \sum_c \mu(c) \sum_{r:\hat{p}(r|c)>z} (\hat{p}(r \mid c) - p(r \mid c)) \right| \end{aligned}$$

By definition $\hat{p}(r \mid c) = p(r \mid c)$ everywhere except for $c \in \mathcal{U}, r \in \mathcal{R}_c$. But for each $c \in \mathcal{U}$, $\hat{p}(c, r)$ is constant over $r \in \mathcal{R}_c$, so $\hat{p}(c, r) > z$ for either all $r \in \mathcal{R}_c$ or none of them. Hence the inner sum above is 0 in any case because $\sum_{r \in \mathcal{R}_c} \hat{p}(r \mid c) - p(r \mid c) = 0$ and $\hat{p}(\bot \mid c) = p(\bot \mid c)$. □

Proofs of Corollary 1 and Theorem 4

With just one correct answer per prompt, like a multiple-choice exam, it is intuitive that one must generate errors if the only valid response is the unique correct answer and one cannot reliably

distinguish correct answers from others. For such a simple case, we show the existence of a threshold t with a better bound. In particular, let

$$\mathrm{err}_{\mathrm{iiv}}(\hat{f}_t) := \Pr_{x\sim D}\left[\hat{f}_t(x) \neq f(x)\right], \text{ where } \hat{f}_t(c,r) := \begin{cases} + & \text{if } \hat{p}(r \mid c) > t, \\ - & \text{if } \hat{p}(r \mid c) \leq t. \end{cases}$$

Hence $\hat{f} = \hat{f}_t$ for $t = 1/\min|\mathcal{E}_c|$ and $\hat{f}$ defined in the paper body. We now state and prove a stronger theorem than Theorem 4. Theorem 4 follows immediately from the definition of $\mathrm{opt}(\mathcal{G})$ and the following theorem.

Theorem 5. *Suppose $|\mathcal{V}_c| = 1$ for all $c \in \mathcal{C}$ and let $C = \min_c |\mathcal{E}_c| + 1$ be the number of choices. Then, for all $p, \hat{p}$, there is some threshold $t \in [0,1]$ such that:*

$$\mathrm{err} \geq 2\left(1 - \frac{1}{C}\right)\mathrm{err}_{\mathrm{iiv}}(\hat{f}_t).$$

Note that the proof of Corollary 1 follows immediately from Theorem 5

Proof of Corollary 1. The proof follows immediately from Theorem 5 and the fact that $\mathrm{err}_{\mathrm{iiv}}(\hat{f}_t) = 1/2$ because a classifier $\hat{f}_t$ based on a trigram model cannot distinguish between c_1, c_2. ☐

We now prove Theorem 5.

Proof of Theorem 5. Consider picking a uniformly random $t \in [0,1]$. We show that:

$$\mathrm{err} \geq 2\left(1 - \frac{1}{C}\right)\mathop{\mathbb{E}}_{t\in[0,1]}[\mathrm{err}_{\mathrm{iiv}}(\hat{f}_t)], \tag{9}$$

This implies that there must exist some threshold $t \in [0,1]$ for which it holds. Note that for uniformly random $t \in [0,1]$,

$$\Pr_{t\in[0,1]}\left[\hat{f}_t(c,r) = +\right] = \hat{p}(r \mid c).$$

First, the expected false positive rate (misclassifications where $\hat{p}(r \mid c) > t$) is:

$$\begin{aligned}\Pr_{t\in[0,1],x\sim D}\left[\hat{f}_t(x) = +, f(x) = -\right] &= \frac{1}{2}\sum_c \mu(c) \sum_{r\in\mathcal{E}_c} \frac{1}{|\mathcal{E}_c|} \Pr_t\left[\hat{f}_t(c,r) = +\right] \\ &\leq \frac{1}{2}\sum_c \mu(c) \sum_{r\in\mathcal{E}_c} \frac{1}{C-1}\hat{p}(r \mid c) \\ &= \frac{1}{2(C-1)}\,\mathrm{err}\,.\end{aligned}$$

Let a_c denote the unique element of $\mathcal{V}_c$. Then the expected false negative rate is,

$$\begin{aligned}\Pr_{t\in[0,1],x\sim D}\left[\hat{f}_t(x) = -, f(x) = +\right] &= \frac{1}{2}\sum_c \mu(c) \Pr_t\left[\hat{f}_t(c,a_c) = -\right] \\ &= \frac{1}{2}\sum_c \mu(c)\,(1 - \hat{p}(a_c \mid c)) \\ &= \frac{1}{2}\,\mathrm{err}\,.\end{aligned}$$

Hence the expected misclassification rate, the sum of the expected false positive and negative rates, satisfies:

$$\mathop{\mathbb{E}}_{t}[\mathrm{err}_{\mathrm{iiv}}(\hat{f}_t)] \leq \frac{1}{2}\left(\frac{1}{C-1}+1\right)\mathrm{err},$$

which is equivalent to Eq. (9) after rearranging terms. □

Supplementary Information References

[1] Good, I. J. The population frequencies of species and the estimation of population parameters. *Biometrika* **40**, 237–264 (1953). URL `https://doi.org/10.1093/biomet/40.3-4.237`.

[2] McAllester, D. & Ortiz, L. Concentration inequalities for the missing mass and for histogram rule error. *Journal of Machine Learning Research* **4**, 895–911 (2003).

[3] McAllester, D. A. & Schapire, R. E. On the convergence rate of Good–Turing estimators. In *Proceedings of the Thirteenth Annual Conference on Computational Learning Theory (COLT 2000)*, 1–6 (Morgan Kaufmann, Palo Alto, California, USA, 2000). URL `https://www.learningtheory.org/colt2000/papers/McAllesterSchapire.pdf`.

[4] McDiarmid, C. On the method of bounded differences. In Siemons, J. (ed.) *Surveys in Combinatorics, 1989: Invited Papers at the Twelfth British Combinatorial Conference*, vol. 141 of *London Mathematical Society Lecture Note Series*, 148–188 (Cambridge University Press, Cambridge, UK, 1989).
